# Quality of life after the initial treatments of non-small cell lung cancer: a persistent predictor for patients’ survival

**DOI:** 10.1186/1477-7525-12-73

**Published:** 2014-05-15

**Authors:** Irawati Lemonnier, Francis Guillemin, Patrick Arveux, Christelle Clément-Duchêne, Michel Velten, Marie-Christine Woronoff-Lemsi, Damien Jolly, Cédric Baumann

**Affiliations:** 1 INSERM, CIC-EC CIC1433, Nancy, France; 2 CHU Nancy, Clinical Epidemiology and Evaluation, Nancy, France; 3 Université de Lorraine, University Paris Descartes, EA 4360 Apemac Nancy, France; 4 Medical Information Department, Georges-François Leclerc Center, Dijon, France; 5 Bourgogne University, EA 4184 Dijon, France; 6 Department of Pulmonology, CHU Nancy, Nancy, France; 7 Department of Epidemiology and Public Health, University of Strasbourg, EA3430 Strasbourg, France; 8 Department of Epidemiology and Biostatistics, Centre Paul Strauss, Strasbourg, France; 9 Clinical Research and Innovation Delegation, University Hospital, Besançon, France; 10 Franche-Comté University INSERM U-1098, Besançon, France; 11 CHU Reims, Robert Debré, Clinical Research and Methodological Unit, F-51092 Reims, France; 12 Faculty of Medicine, Champagne-Ardenne Reims University, EA 3797, F-51095 Reims, France

**Keywords:** Quality of Life, SF-36, QLQ C-30, Non-small-cell lung cancer, Survival

## Abstract

**Background:**

Health-related quality of life (HRQoL) before treatment may predict survival of patients with non-small-cell lung cancer (NSCLC). We investigated the predictive role of HRQoL after the initial treatments, on the survival of these patients.

**Methods:**

A prospective multi-center study conducted in northeastern France. The SF-36 and European Organization for Research and Treatment of Cancer, Quality of Life Questionnaire Core-30 (QLQ C-30) were mailed to patients 3 months after the end of the diagnostic process. High scores for functioning dimensions on both questionnaires indicated better QoL, and low scores for symptom dimensions on the QLQ C-30 indicated few symptoms. Cox regression modeling was used to identify predictive factors of survival.

**Results:**

In total, 230 (63.5%) patients responded to the SF-36 and QLQ C-30. Before completing the questionnaires, almost 60% of patients had undergone some chemotherapy, about 10% underwent radio/chemotherapy or both and more than 30% underwent surgery or surgery plus chemo/radiotherapy.

On SF-36, the highest mean score was for social functioning dimension (55.5 ± 28), and the lowest was for the physical role dimension (17.9 ± 32.2).

On QLQ C-30, for the functioning dimensions, the highest mean score was for cognitive functioning (74.6 ± 25.9) and the lowest was for role functioning (47.2 ± 34.1). For symptom dimensions, the lowest score was for diarrhoea (11.5 ± 24.2) and the highest was for fatigue (59.7 ± 27.7).

On multivariate analysis, high bodily pain, social functioning and general health scores (SF-36) were associated with a lower risk of death (hazard ratio 0.580; 95% confidence interval [0.400–0.840], p = 0.004; HR 0.652 [0.455–0.935], p < 0.02; HR 0.625 [0.437–0.895] respectively). Better general QoL on QLQ C-30 was related to lower risk of death (HR 0.689 [0.501–0.946], p = 0.02).

**Conclusion:**

Adding to previous knowledge about factors that may influence patients QoL, this study shows a persisting relationship between better perceived health in HRQoL after the initial treatment of NSCLC and better survival.

## Introduction

Lung cancer is considered the greatest contributor to death from cancer. It accounts for 1,180,000 deaths per year worldwide [1] and in France 26,624 deaths in 2005. In France, its incidence and mortality rates have decreased by 0.5% and 1.7%, respectively, since 1980 for men, but have increased for women [2]. Non-small-cell lung cancer (NSCLC) is accounted for 80% of these new cases [3,4] and survival time for all stages of NSCLC (I, II, III-IV) is low with a median of 43.3 months [4]. Therefore, all prognostic factors must be identified in this group of patients to provide insights into the disease process and the therapeutic response.

Patient-reported outcomes, including health-related quality of life (HRQoL), symptoms, and functional status, are well established and useful in oncology for describing the clinical disease course, helping to select optimal treatment, or comparing populations of cancer patients with those having other diseases and with the general population [5-9]. In general, HRQoL covers subjective perceptions of the positive and negative aspects of patient symptoms, including physical, emotional, social and cognitive functions, and importantly, disease symptoms and side effects of treatments [10]. Clinicians are increasingly considering HRQoL as critical to cancer patient care [11,12].

Different instruments have been developed and validated [13,14], and recently, researchers have begun to study the relation between HRQoL and patient’s survival. Often, patient HRQoL is measured after cancer diagnosis and before any treatment, and studies have shown different results. For example, measuring by the European Organization for Research and Treatment of Cancer (EORTC), Quality of Life Questionnaire Core-30 (QLQ C30), global HRQoL assessed before treatment, was found to be a strong predictor of survival in patients with NSCLC and lymph node abnormalities [15]. With this same instrument, another study showed that patient self-reported pain and dysphagia predicted overall survival in advanced NSCLC, independently of socio-demographic or clinical characteristics [16]. Fielding *et al*. used the Functional Assessment of Cancer Therapy-General instrument to measure patients with liver and lung cancer HRQoL in China and found that only physical well-being subscale predicted lung cancer survival [17]. However, Hernsdon *et al*. showed that after adjusting for clinical factors, the pain perceived by patients predicted survival, whereas overall HRQoL of the QLQ C-30 did not [18].

These heterogeneous results indicate that more research is needed to better understand the role of patient’s QoL on survival. In particular, the diagnostic process ending with delivery of the diagnosis and the treatment strategy that can be sources of psychological challenge [19,20], and the early period of treatment that may add potential distress. Measuring HRQoL within months after the end of the diagnosis process reflects both the effect of the disease evolution and of the treatments during this period. A 3-month time frame period after the diagnosis process gives enough time to patients to undertake and receive some benefits from their treatments and at the same time to avoid a selection bias due to loosing too many patients. Whether the treatment received has levelled off the predictive value of HRQoL on survival remains unknown. We aimed to investigate whether HRQoL after the initial treatment still plays a role in predicting survival in patients with NSCLC. We used the Medical Outcomes Study Short Form 36 (SF-36) and the QLQ C-30 to evaluate HRQoL. The SF-36 has been useful in surveys of general and specific populations comparing the relative burden of diseases and differentiating the health benefits produced by a wide range of treatments [14,21]. The QLQ C-30 allowed identifying health problems related to cancer and its treatments, such as pain, diarrhoea, nausea and vomiting, as well as appetite loss [13,15].

## Methods

### Design

A prospective multi-center study was conducted in northeastern France (Alsace, Lorraine, Franche-Comté, Bourgogne, and Champagne-Ardenne) as part of research reported elsewhere [22-24]. The study involved 18 health-administrative districts covering 8.22 million people. Fifty physicians in 18 principal hospitals, public or private, in each region participated and reported the cases of lung cancer in their hospital occurring from July 2002 to June 2006. The study was approved by the Institutional Review Board (Commission National d’Informatique et Liberté [CNIL]).

### Samples

The inclusion criteria were patients with NSCLC; were at least 18 years old; underwent the pre-therapeutic process within the participating regions; began their treatment < 10 weeks before inclusion; were able to read and understand French, and to complete self-reported questionnaires.

### Data collection

For each reported case, a research assistant in each region verified the patient’s medical record and noted the patient’s sociodemographic characteristics (age, sex, and level of education), stage of cancer, health-examinations that patient undertook during the diagnostic process, and all treatment (radiotherapy, chemotherapy or surgery) undertook after the end of the diagnosis process until the HRQoL measure.

Information on patients’ vital status at the end of follow-up was obtained by accessing the Repertoire National d’Identification des Personnes Physiques, a register of death certificates with documented medical cause (CNIL authorization n° 908413, November 2008).

#### HRQoL measure

The date when the physician decided to stop diagnostic investigations or begin treatment was considered as the end of the diagnostic process. HRQoL was assessed by the generic (SF-36) and cancer-specific (QLQ C-30) self-administered questionnaires mailed 10 (+2) weeks after this date.

The SF-36, a multipurpose health survey with 36 questions, measures HRQoL status during the previous 4 weeks. Scores range from 0 to 100, with high scores reflecting better QoL. The survey yields scores for 8 scales or dimensions of functional health and well-being: physical functioning, physical role, bodily pain, general health, vitality, social functioning, emotional role and mental health. The questionnaire allows for a psychometrically based *physical component summary* (PCS) that measures the absence of physical limitations, disability or decrease in well-being and energy level and a *mental component summary* (MCS) that measures the absence of psychological distress and limitations in usual social or role activities because of emotional problems during the last 8 days.

The cancer-specific instrument was the QLQ C-30. The survey contains 30 questions addressing various aspects of HRQoL. Following EORTC guidelines, subscale scores were converted to a 0–100 scale. High scores represent a better level of functioning for the functional and global health status and more severe symptoms for the symptom scales. The reliability and validity of this questionnaire have been confirmed in a number of international studies for patients with different cancers, including lung cancer [13,15]. It measured the HRQoL of patients during the week before the measure.

The generic and cancer-specific questionnaires were complementary.

### Statistical analysis

The analysis was done on data of patients who returned back HRQoL measures (SF-36 and QLQ-C30) and on which the scores could be calculated. Questionnaires with missing data were handled according to imputation method recommended by the developers.

#### Descriptive analysis

HRQoL scores were described with means ± SD, median and quartile 1 (Q1) and Q3. To test the association between variables, the chi-square or Fisher exact test was used for qualitative variables and Student *t* test or Mann–Whitney test for quantitative variables.

#### Survival analysis

Using survival as the outcome, the time to event (death) was determined from the date when patients completed the HRQoL questionnaire to the date of death (due to any cause = overall survival). Patients who were alive on May 1, 2010 or were lost to follow-up were censored.

Survival curves were estimated by the Kaplan-Meier method and compared by the log rank test. Bivariate analyses involved use of the Cox proportional hazards model to identify HRQoL dimensions and patient characteristics related to survival: age (as a continuous variable), sex, cancer stage, and treatment. The proportionality assumption was checked for each of the variables under study with scaled Schoenfeld residuals and by the proportionality test [25-27]. According to Van Steen *et al*. [28], global HRQoL score is highly correlated with 7 of 11 scores of the QLQ C-30, and the authors suggested excluding this variable from the final model when analyzing prognostic factors. In addition, both SF-36 summaries are highly correlated with 4 of the scale’s 10 dimensions [14]. The PCS is related to physical functioning, role physical, bodily pain and general health dimensions and the MCS to vitality, social functioning, role emotional and mental health [29]. Accordingly, we built several models on multivariate analysis using the Cox model. Survival was used as independent variable, and certain dimensions on the SF-36 and QLQ C-30 that were statistically significant on bivariate analysis were used as candidate predictors as explained below. We also tested first-order interactions between age, sex, stage of cancer and treatment before QoL measure. Significant interactions were included in the models.

### Models with SF-36 scores

Model 1: All HRQoL dimension scores except global health, PCS, and MCS scores.

Model 2: General health score only.

### Models with QLQ C-30 scores

Model 4: All HRQoL dimension scores except global HRQoL score.

Model 5: Global HRQoL score only.

Each model was adjusted on patients’ age and sex, cancer stage and treatments before HRQoL measurement. Treatment was classified into 3 classes: 1) radiotherapy and chemotherapy, 2) surgery alone or surgery and (radiotherapy or chemotherapy), and 3) chemotherapy only.

The multivariate analysis involved all variables that were significantly related to survival on bivariate analysis at p < 0.1. A p < 0.05 was considered statistically significant for all analyses. Analyses involved use of SAS 9.3 (SAS Inst., Cary, NC).

## Results

### Patient characteristics

**Figure 1:**
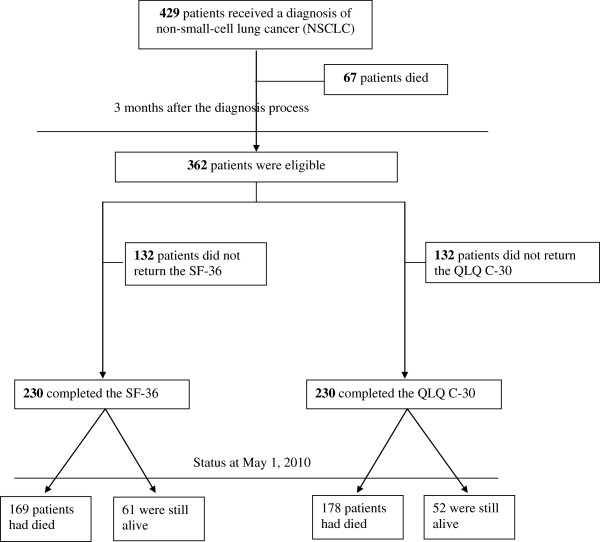
**Patients with non-small-cell lung cancer (NSCLC) who participated in the quality-of-life study 3 months after the diagnostic process.** SF-36 = Medical Outcomes Survey Short form 36; QLQ C-30 = European Organization for Research and Treatment of Cancer Quality of Life Questionnaire Core-30.

Among 429 NSCLC patients identified for the study, 67 patients died before we distributed the questionnaire 3 months after the diagnostic process. Of 362 potential participants, 230 (63.5%) returned back and completed the SF-36 and the QLQ C-30 (Figure 1). Patients who died before 3 months were older (mean 64.2 ± 1.3 years; p = 0.02) and more often had stage 4 NSCLC (p = 0.002) than patients who were still alive and could participate in the study. Before completing the questionnaires, most participating patients had received some treatments: almost 60% received chemotherapy only during 82.5 ± 61.3 days, about 11% had some chemo and radiotherapy; and more than 30% underwent surgical treatment and chemo/radiotherapy (Table 1). The mean delay between the beginning of treatment and when patients completed the questionnaires was 71.9 ± 34.2 days.

**Table 1:** Characteristics of patients with non-small-cell lung cancer (NSCLC) who completed the SF-36 and QLQ C-30 health-related quality of life (HRQoL) surveys

	**SF-36 (n = 230)**		**QLQ C-30 (n = 230)**	
	**Mean ± SD**	**n [%]**	**Mean ± SD**	**n [%]**
Age	60.2 ± 10.7		60.1 ± 10.7	
Sex:				
- Men		186 [80.9]		184 [80.0]
- Women		44 [19.1]		46 [20.0]
Stage of cancer				
- I or II		49 [21.3]		48 [20.9]
- III		136 [59.1]		141 [61.3]
- IV		45 [19.6]		41 [17.8]
Treatments before the HRQoL measurement				
- Chemotherapy only		133 [57.8)		132 [57.4]
- Radiotherapy only or with chemotherapy		26 [11.3]		24 [10.4]
- Surgery alone or surgery and (radiotherapy or chemotherapy)		71 [30.9]		74 [32.2]
Education level				
- Primary		64 [28.7]		64 [29.6]
- Secondary		144 [64.6]		136 [63.0]
- Post-secondary		15 [6.7]		16 [7.4]

SF-36 = Medical Outcomes Survey Short Form 36; QLQ C-30 = European Organization for Research and Treatment of Cancer Quality of Life Questionnaire Core-30.

Treatments before HRQoL measurement were divided into 3 classes for all Cox multivariate models:

1) Chemotherapy only.

2) Radiotherapy or chemotherapy or both.

3) Surgery alone or surgery and radiotherapy or chemotherapy.

No difference between responders (n = 230) vs non-responders (n = 132) was observed for: age, p = 0.6; sex, p = 0.9; education level, p = 0.5; treatments, p = 0.08, stage, p = 0.5. Neither for the median survival of both groups (15.5 months [CI 95% = 10 – 30] for patients non-responders the questionnaires, and 18.5 months [14 – 27] for responders.

At the end of the follow-up (up to 8 years; mean 32 ± 29.6 months), 70% of responders had died.

### HRQoL scores 3 months after the diagnostic process

#### SF-36

The highest mean scores were for the physical- and social-functioning dimensions and for mental health (50.1 ± 27.2, 55.5 ± 28 and 52.5 ± 21.6, respectively), and the lowest score was for the physical role dimension (17.9 ± 32.2) - Table 2.

**Table 2:** Description of SF-36 scores at 3 months of follow up

	**N**	**Mean**	SD*	**Median**	**Q1**	**Q3**
**Physical functioning**	230	50.1	27.2	50	27.8	70
**Physical role**	229	17.9	32.2	0	0	25
**Bodily pain**	229	46.2	25	41	31	62
**Mental health**	228	52.5	21.6	52	36	68
**Emotional functioning**	226	24.6	38	0	0	33.3
**Social functioning**	230	55.5	28	50	37.5	75
**Vitality**	227	32.9	19.6	30	20	45
**General health**	230	39.5	19.3	37	25	52
**PCS**	225	34.8	8.4	34.7	28.2	40.9
**MCS**	225	37.2	11.3	35.7	28.9	45.1

*SD = Standard deviation.

#### QLQ C-30

For the functioning dimensions, the highest mean score was for cognitive functioning (74.6 ± 25.9) and the lowest was for role functioning (47.2 ± 34.1). For symptom dimensions, the lowest score was for diarrhoea (11.5 ± 24.2) and the highest was for fatigue (59.7 ± 27.7) – Table 3.

**Table 3:** Description of QLQ C-30 scores at 3 months of follow up

	**N**	**Mean**	SD*	**Median**	**Q1**	**Q3**
**Physical functioning**	230	63.2	23.9	66.7	53.3	80
**Role functioning**	228	47.2	34.1	50	16.7	66.7
**Cognitive functioning**	230	74.6	25.9	83.3	66.7	100
**Emotional functioning**	230	58.6	28.2	66.7	33.3	83.3
**Social functioning**	229	55.5	33.7	66.7	33.3	83.3
**Fatigue**	230	59.7	27.7	55.6	33.3	77.8
**Pain**	230	43.5	33.5	33.3	16.7	66.7
**Nausea and vomitting**	230	20.6	29.1	0	0	33.3
**Insomnia**	229	44.3	37.2	33.3	0	66.7
**Constipation**	228	28.1	33.9	33.3	0	33.3
**Dyspnea**	230	55.5	34.5	66.7	33.3	100
**Diarrhea**	229	11.5	24.2	0	0	0
**Appetit loss**	229	40	38.3	33.3	0	66.7
**Global QoL**	230	45.6	20.2	50	33.3	58.3

*SD = Standard deviation.

There were few (from 1 to 5) missing data in both questionnaires (see the N on Tables 2 and 3).

### Prognostic value of HRQoL scores for patient survival

#### Bivariate analysis

Disease stage and treatments independently predicted survival (Table 4). Patients with stage III and those with stage IV have much more than double risks of death (p < 0.001 to p = 0.02 respectively) compared to patients with stage I or II. Patients treated by surgery alone or surgery with chemo/radiotherapy before QoL measure was associated with a lower risk of death (p < 0.001). On the SF-36, scores ≥50 of bodily pain, emotional role, social functioning and vitality (p = 0.001 to p = 0.03; Table 4), independently predicted a better survival. Score ≥50 on general health was also related to a better survival (HR 0.625 [0.437 – 0.895], p = 0.01) – Table not shown. On the questionnaire QLQ C-30, scores ≥50 of the function domains except the cognitive- and emotional-functioning were also related to a better survival (physical-, role- and social-functioning; p = 0.009 to p = 0.005; Table 5); and scores <50 of 4 of 8 symptoms’ (less fatigue, constipation, nausea/vomiting, and appetite loss; p = 0.004 to p = 0.01), independently predicted higher risk of death. Scores ≥50 on general QoL were also related to a better survival (Table 5).

**Table 4:** The prognostic effect of SF-36 HRQoL dimensions 3 months after NSCLC diagnosis on survival (bivariate and multivariate analyses)

**Model without general health**						
**Variables**	**Bivariate analysis**			**Multivariate analysis**		
	**HR**	**95% CI**	**P**	**HR**	**95% CI**	**p**
**HRQoL dimensions**						
Physical functioning ≥50	0.750	0.550 - 1.023	0.069	0.946	0.667 - 1.341	0.755
Physical role ≥50	0.651	0.428 - 0.991	0.046	0.576	0.326 - 1.016	0.057
Bodily pain ≥50	0.590	0.430 - 0.810	0.001	0.580	0.400 - 0.840	0.004
Emotional role ≥50	0.665	0.452 - 0.980	0.039	1.564	0.923 - 2.648	0.096
Social functioning ≥50	0.676	0.489 - 0.933	0.017	0.652	0.455 - 0.935	0.020
Mental health ≥50	0.792	0.581 - 1.081	0.142			
Vitality ≥50	0.605	0.405 - 0.905	0.015	0.784	0.472 - 1.303	0.349
**Patient characteristics**						
Age	1.002	0.9 - 1.02	0.798	1.102	1.041 - 1.167	<0.001
Sex:						
- Men	1			1		
- Women	0.859	0.575 - 1.285	0.460	0.556	0.351 - 0.881	0.012
Education level:						
- Primary	1					
- Secondary	0.820	0.581 - 1.157	0.259			
- Post secondary	0.739	0.374 - 1.461	0.384			
Stage of cancer						
- Stage I - II	1			1		
- Stage III	1.670	1.088 - 2.565	0.019	21.388	3.460 - 132.187	0.001
- Stage IV	3.341	2.025 - 5.511	<0.001	633.964	21.799 - 18437.11	<0.001
Treatment before the HRQoL measurement						
- Chemotherapy only	1			1		
- Chemo and radiotherapy	0.691	0.420 - 1.138	0.146	0.651	0.387 - 1.095	0.106
- Surgery alone or surgery and radiotherapy or chemotherapy	0.219	0.144 - 0.333	<0.001	0.186	0.115 - 0.300	<0.001
**Interaction**						
Age * Stage of cancer	0.969	0.945 - 0.994	0.016	0.952	0.926 - 0.978	<0.001

*The interaction between Age and Stage of cancer.

HR = hazard ratio; 95% CI = 95% confidence interval.

Scores range from 0 to 100, with high scores reflecting better QoL.

**Table 5:** The prognostic effect of QLQ C-30 variables 3 months after NSCLC diagnosis on survival (bivariate and multivariate analyses)

**Model without global health**						
	**Bivariate analysis**			**Multivariate analysis**		
**Variables**	**HR**	**95% CI**	**p**	**HR**	**95% CI**	**p**
**Functioning domains (100 = good)**						
Physical functioning ≥50	0.612	0.431 - 0.869	0.006	0.787	0.506 - 1.226	0.289
Role functioning ≥50	0.640	0.469 - 0.874	0.005	0.907	0.573 - 1.437	0.677
Cognitive functioning ≥50	0.916	0.554 - 1.152	0.733			
Social functioning ≥50	0.656	0.477 - 0.900	0.009	1.058	0.695 - 1.609	0.794
Emotional functioning ≥50	0.841	0.604 - 1.170	0.304			
**Symptom domains (100 = severe)**						
Pain <50	0.774	0.567 - 1.056	0.106			
Fatigue <50	0.616	0.445 - 0.851	0.003	0.703	0.471 - 1.049	0.084
Sleep disturbance <50	0.876	0.641 - 1.197	0.406			
Constipation <50	0.660	0.460 - 0.948	0.024	0.786	0.523 - 1.181	0.246
Nausea and vomiting <50	0.624	0.434 - 0.897	0.011	0.849	0.529 - 1.363	0.498
Apetite loss <50	0.628	0.457 - 0.863	0.004	0.890	0.599 - 1.324	0.567
Diarhoea <50	0.660	0.373 - 1.168	0.153			
Dyspnea <50	1.095	0.803 - 1.491	0.567			
**Patient characteristics**						
Age	1.011	0.979 - 1.044	0.497	1.085	1.023 - 1.152	0.007
Sex:						
- Men	1			1		
- Women	0.732	0.293 - 1.831	0.505	0.633	0.411 - 0.976	0.038
Education level:						
- Primary	1					
- Secondary	0.783	0.554 - 1.107	0.167			
- Post secondary	0.695	0.352 - 1.372	0.294			
Stage of cancer						
- Stage I - II	1			1		
- Stage III	1.971	0.916 - 4.239	0.011	13.463	2.036 - 89.038	0.007
- Stage IV	4.898	1.518 - 15.801	<0.001	207.281	6.471 - 6639.251	0.003
Treatment before the HRQoL measurement						
- Chemotherapy only	1			1		
- Chemo and radiotherapy	0.656	0.388 - 1.109	0.115	0.635	0.366 - 1.103	0.107
- Surgery alone or surgery and radiotherapy or chemotherapy	0.229	0.152 - 0.345	<0.001	0.227	0.143 - 0.363	<0.001
**Interaction**						
Age*stage of cancer	0.973	0.947 - 1.000	0.049	0.959	0.932 - 0.987	0.004
**Model with global health only**						
	**Bivariate analysis**			**Multivariate analysis**		
**Variables**	**HR**	**95% CI**	**p**	**HR**	**95% CI**	**p**
**(100 = good)**						
Global HRQoL ≥50	0.688	0.504 - 0.938	0.018	0.689	0.501 - 0.946	0.021
**Patient characteristics**						
Age	1.011	0.979 - 1.044	0.497	1.083	1.022 - 1.147	0.007
Sex:						
- Men	1			1		
- Women	0.732	0.293 - 1.831	0.505	0.682	0.452 - 1.029	0.068
Education level:						
- Primary	1					
- Secondary	0.783	0.554 - 1.107	0.167			
- Post secondary	0.695	0.352 - 1.372	0.294			
Stage of cancer						
- Stage I - II	1			1		
- Stage III	1.971	0.916 - 4.239	0.011	11.982	1.881 - 76.310	0.009
- Stage IV	4.898	1.518 - 15.801	<0.001	167.961	5.666 - 4979.331	0.003
Treatment before the HRQoL measurement						
- Chemotherapy only	1			1		
- Chemo and radiotherapy	0.656	0.388 - 1.109	0.115	0.690	0.406 - 1.173	0.171
- Surgery alone or surgery and radiotherapy or chemotherapy	0.229	0.152 - 0.345	<0.001	0.239	0.151 - 0.377	<0.001
**Interaction**						
Age*stage of cancer	0.973	0.947 - 1.000	0.049	0.962	0.935 - 0.989	0.007

*The interaction between Age and Stage of cancer.

*HR* = hazard ratio; *95% CI* = 95% confidence interval.

### Survival analysis

The comparison of patients’ survival according to the HRQoL scores showed that:

– On SF-36,

patients with Bodily pain (BP) scores <50, the median survival was 9 months (CI 95%: 7 – 16) compared to 35.5 (26 – 57) if scores were ≥ 50, p < 0.001;

for Social functioning, those with score <50 had a median of 8 months (5 – 25) vs 25.5 (15 – 37), p = 0.01 in patients with score ≥50;

for Global health <50 the median survival was 14 (8–20) vs 37 (20 – 58) if scores ≥ 50.

– On QLQ C-30, patients with the general QoL score <50, had a median survival of 9 months (7 – 17) compared to 29.5 (9 – 45) if scores ≥ 50.

#### Multivariate analysis

##### SF-36

1) For the model without general health scores, higher bodily pain and social functioning scores (≥50) was associated with a lower risk of death (hazard ratio [HR] 0.589, 95% confidence interval [0.400–0.840], p = 0.05 and HR = 0.652 [0.455-0.935], p = 0.004).

2) For the model with the general health score alone, higher score (≥50) on this domain was also associated with lower risk of death (HR = 0.625 [0.437-0.895], p = 0.01).

##### QLQ C-30

1) For models without global HRQoL, no association was observed between HRQoL dimensions and patients’ survival.

2) For the model with global HRQoL only, higher global HRQoL score (≥50) was associated with lower risk of death (HR = 0.689 [0.501-0.946], p = 0.02) (Table 5).

For all models, among socio-demographic or clinical characteristics: older age, stage III and IV were related to higher risk of death. Surgery and radio/chemotherapy as treatment was related to a decreased risk of death. Older patients with stage 4 had a lower risk of death than younger patients with the same stage of cancer (age by stage interaction HR = 0.9 [0.9 – 0.9]; p < 0.001) (Tables 4, and 5).

## Discussion

This study showed that certain domains on health related quality of life, measured after the initial treatment, were related to the survival of patients with non-small-cell lung cancer. On the bivariate analysis, we observed that better physical- and emotional-role, social functioning and vitality as well as lesser pain symptom on SF-36 (score ≥50) were related to a better patients’ survival. On multivariate analysis, a persistent relation was observed for 2 domains: lesser (high scores) Bodily pain and better Social-functioning. The QLQ C-30 showed that on the bivariate analysis, better (high scores) physical-, role- and social functioning, and also global HRQoL as well as lesser symptoms (low scores) on fatigue, constipation, nausea-vomiting and appetite loss were related to a better survival. On the multivariate analysis, only a better Global QoL found to be related to a better patient’s survival. This relation was observed independently of known prognostic variables i.e. age, sex, stage of cancer, and initial treatments. Patients seemed to live few months longer with better perceived health.

This multicenter, prospective study involved patients with a wide variety of NSCLC severity (stage I to IV) while they were undergoing treatments. With a follow-up of 4 to 6 years, we could observe patient’s survival and whether HRQoL during treatments period continued to be related to survival. It is well known that both questionnaires that were used, the SF-36 and QLQ-C30, had robust psychometric properties. Also that HRQoL is one of measurements that revealed patients’ point of view regarding his/her health status and encompass the incidence and the extent of limitations due to physical capacity, which might occur because of the disease itself or the effects of treatment. Indeed, as stated by Franceshini *et al*., 54% of patients with lung cancer report dyspnoea, which contributes to the worsening of QoL, because this symptom might limit the ability to perform activities of daily living and to work [30].

Studies have shown different results on the relation between sex and survival of patients with NSCLC. The present study confirmed findings that women had a better survival [31-34].

Readers need to be cautious in translating the results of this study to other populations. First, we measured HRQoL 3 months after the diagnostic process, so it represented patients’ perceptions of the overall early management of lung cancer, from diagnosis to therapy. However, this time period may introduce some selection bias because some patients had died or were lost to follow-up before HRQoL measurement. Patients who did not make it until a 3 month follow-up were older and mostly had a stage 4 of cancer. Among potentials responders, more than half (63.5%) returned back completed questionnaires, with few (from 1 to 5) missing data. Also, there was neither socio-demographical nor clinical characteristics difference between responders vs non responders. Second, previous studies showed poor performance status associated with worse survival and good performance with better survival [15,16,35]. We had information on treatments that were performed after the HRQoL measurement and we assumed, according to standards, that these treatments were prescribed in accordance with each patient’s condition, integrating performance status. Also, we did not dispose of information regarding the stage of treatment and therefore could not confirm the hypothesis that this variable may relate to survival.

Previous studies showed that better HRQoL scores measured at baseline (before any treatment) related to better survival of patients with cancer [15,16,36,37]. This study confirmed previous ones that reported that better global QoL (QLQ C-30) as the strongest predictor of better survival [15,36,37]. However it did not find perceived pain as an independent predictor, as showed by another study for patients with stage III or IV NSCLC measured at diagnosis [16]. In the present study, we measured HRQoL 3 months after the diagnosis, when cancer management and treatments were ongoing. The mean scores on the SF-36 were increased by +0.5 to +6 points for patients who had some surgical treatment (n = 74) before HRQoL measurement than for those who did not. The results of our study suggested that, in addition to previous studies showing baseline HRQoL affecting survival, perceived lesser-pain and better social functioning measured by the SF-36 and perceived general QoL on QLQ C-30 at 3 months after the diagnosis may still affect survival. The better the HRQoL scores, the better the survival probability. As suggested by Gotay et.al., changes in HRQOL may be an early warning system that can be useful for clinical decision making, because HRQOL may deteriorate before disease progression is evident by other measures [36]. Although the observed impact of HRQoL may not be that strong, it plays a very important role besides other factors known that can influence these patients’ survival. However, further studies are needed to explore the fluctuation over time of HRQoL and whether this fluctuation, if it exists and depending on its time of measurement, is related to patient survival.

## Conclusion

Measured after the initial treatment, this study showed a persistent relation between HRQoL and survival of patients with non-small lung cancer. Lesser perceived pain, a better perception on general health and general QoL are found to be related to a better chance to survive few months longer. This relation was observed independently of known prognostic variables i.e. age, sex, stage and initial treatments. Our results have implications for clinical practice. Clinicians may integrate these measures in their battery of indicators to continue monitoring patient health after the beginning of treatment and to carefully examine symptoms in self-rated HRQoL assessment in order to improve patients’ survival. If used in routine practice, it may also be useful to facilitate communication among clinicians, health professionals and patients to identify the most adaptable cancer management.

### Consent

No image was taken and patients gave their consent to publish the results of our study.

## Competing interests

The authors declare that they have no competing interests.

## Authors’ contributions

Study concepts: FG, DJ, PA, MV, MC-WL. Study design: FG, DJ, PA, MV, MC-WL. Data acquisition: FG, DJ, PA, MV, MC-WL, IL. Quality control of data and algorithms: FG, DJ, PA, MV, MC-WL, IL, CB. Data analysis and interpretation: FG, DJ, PA, MV, MC-WL, IL, CCD, CB. Statistical analysis: IL, CB. Manuscript preparation: IL, CB, FG. Manuscript editing: FG, DJ, PA, MV, MC-WL, IL, CCD, CB. Manuscript review: FG, DJ, PA, MV, MC-WL, IL, CCD, CB. All authors read and approved the final manuscript.
